# Muonium in Stishovite: Implications for the Possible Existence of Neutral Atomic Hydrogen in the Earth's Deep Mantle

**DOI:** 10.1038/srep08437

**Published:** 2015-02-13

**Authors:** Nobumasa Funamori, Kenji M. Kojima, Daisuke Wakabayashi, Tomoko Sato, Takashi Taniguchi, Norimasa Nishiyama, Tetsuo Irifune, Dai Tomono, Teiichiro Matsuzaki, Masanori Miyazaki, Masatoshi Hiraishi, Akihiro Koda, Ryosuke Kadono

**Affiliations:** 1 Department of Earth and Planetary Science, University of Tokyo, Tokyo 113-0033, Japan; 2 Muon Science Laboratory, Institute of Materials Structure Science, High Energy Accelerator Research Organization, Tsukuba 305-0801, Japan; 3 Department of Earth and Planetary Systems Science, Hiroshima University, Higashi-Hiroshima 739-8526, Japan; 4 National Institute for Materials Science, Tsukuba 305-0044, Japan; 5 Geodynamics Research Center, Ehime University, Matsuyama 790-8577, Japan; 6 Earth-Life Science Institute, Tokyo Institute of Technology, Tokyo 152-8550, Japan; 7 Nishina Center for Accelerator-Based Science, RIKEN, Wako 351-0198, Japan

## Abstract

Hydrogen in the Earth's deep interior has been thought to exist as a hydroxyl group in high-pressure minerals. We present Muon Spin Rotation experiments on SiO_2_ stishovite, which is an archetypal high-pressure mineral. Positive muon (which can be considered as a light isotope of proton) implanted in stishovite was found to capture electron to form muonium (corresponding to neutral hydrogen). The hyperfine-coupling parameter and the relaxation rate of spin polarization of muonium in stishovite were measured to be very large, suggesting that muonium is squeezed in small and anisotropic interstitial voids without binding to silicon or oxygen. These results imply that hydrogen may also exist in the form of neutral atomic hydrogen in the deep mantle.

Hydrogen is the most abundant element in the solar system. It binds to oxygen and the resultant water makes the Earth a habitable blue planet. Ocean covers 70% of the Earth's surface. Moreover, a significant amount of water may be hidden in the Earth's interior. The water in anhydrous silicates is considered to be related to lattice defects and incorporated into the system, for example, by simultaneous substitutions such as Mg^2+^ → H^+^ and O^2−^ → OH^−^ (Ref. 1). If a structural change is induced by significant substitutions, the system should be classified in hydrous silicates. In the field of earth science, the water in anhydrous silicates has been studied mainly by the methods to probe the oscillation of hydroxyl group, such as infrared absorption and Raman scattering^2^,^3^,^4^,^5^. It has been reported that the mantle-transition zone can accommodate a significant amount of H_2_O component up to several times as much as the total mass of ocean^1^ and, although it is not a consensus^4^, the lower mantle could also accommodate a non-negligible amount^3^.

At the same time, one can speculate that hydrogen might exist in silicates without substitutions. In the field of applied physics, hydrogen in perfect crystals of low-pressure phases of SiO_2_ has been studied by the methods to probe H more directly^6^,^7^,^8^,^9^,^10^, such as Muon Spin Rotation (μSR) and Electron Paramagnetic Resonance (EPR), to obtain a better understanding of Metal-Oxide-Semiconductor (MOS) devices. In those studies, it has been reported that hydrogen can exist in interstitial voids of structure. Moreover, it has recently been revealed that a significant amount of small molecules, such as H_2_ and He, dissolves into interstitial voids of low-pressure phases of SiO_2_ (Refs. 11,12,13,14,15). Therefore, it seems possible that the hydrogen which is not directly related to lattice defects of silicates and has not so far been recognized sufficiently in earth science may exist in the mantle. However, because interstitial voids of low-pressure phases of SiO_2_ are large, the above findings could be exceptional. The structures of two important SiO_2_ minerals, stishovite and quartz, are compared in Figure 1. Stishovite is a rutile-type high-pressure phase and quartz is an ambient-pressure phase. The two structures show marked contrast in interstitial voids, in addition to the well-known difference in coordination number.

Here, we present the results of μSR spectroscopy to show the possible existence of neutral atomic hydrogen in small and anisotropic interstitial voids of stishovite. Although μSR spectroscopy is not very popular in earth science, it is a well-established technique to simulate the behavior of hydrogen in semiconductors and insulators^16^,^17^. Positive muon can be considered as a light isotope of proton with a mass of about 1/9 that of proton. In μSR, spin-polarized positive muons are implanted into matter and their subsequent behaviors (changes in spin polarization with time) are measured under various conditions of magnetic field. μSR is a powerful tool to probe the position, electronic state, and other information of the hydrogen that exists irregularly in matter, because it tracks the behavior of each implanted particle (in contrast to diffraction). In this study, μSR was conducted on powder and pellet samples to clarify intrinsic properties of stishovite (see Methods).

## Results

An example of the time evolution of muon-spin polarization in stishovite powder is shown in Figure 2a. These spectra were measured at a transverse field of 350 G (nominal) at TRIUMF (Canada). The upper and lower limits of the vertical axis of the graph correspond to the amplitude for fully spin-polarized muons. This figure clearly indicates that the fraction of muon staying in the initial diamagnetic state (corresponding to OH forming hydrogen) is small. The muon fraction in stishovite increased slightly with decreasing temperature; 14% at 300 K, 14% at 250 K, 15% at 200 K, 16% at 150 K, 17% at 100 K, 17% at 50 K, and 19% at 2.5 K in stishovite powder.

The spectrum at 300 K in Figure 2a is enlarged in terms of the horizontal axis and is compared with quartz in Figure 2b,c. High-frequency oscillation of muonium-spin polarization is seen. The fraction of muonium (= muon-electron bound paramagnetic state, corresponding to neutral atomic hydrogen) can be determined from the amplitude of oscillation at time = 0 after subtracting the contribution of muon-spin polarization. Only the triplet state, in which muon and electron have spins of the same direction, appears in these spectra and the singlet state, in which they have spins of the opposite direction, has the same population as the triplet and does not appear; twice the amplitude of the triplet state corresponds to the fraction of muonium^16^,^17^. It was determined to be 56% in stishovite at 300 K. The sum of muon and muonium fractions is smaller than 100% (Table 1). The remaining 30% was missing due to fast relaxation in spin polarization. These spectra also show that the relaxation rate of muonium-spin polarization in stishovite is large.

The beat of oscillation due to the splitting of the triplet state under transverse field is also seen in Figure 2b,c. The frequencies determined from the spectrum for stishovite are 452(10) and 562(32) MHz. The hyperfine-coupling parameter of muonium A/h, where h is the Plank constant, can be determined from these frequencies. A/h's for powder and pellet samples agree well with each other (Table 1). A/h for muonium in stishovite at 300 K is determined to be 4.67(3) GHz with all the spectra for the two samples at transverse fields from 200 to 1000 G (nominal). A/h at 2.5 K is 5.17(14) GHz, estimated only from the two frequencies at 350 G (nominal). These values are significantly larger than 4.49(2) GHz in quartz (Table 1) and 4.463 GHz in vacuum^18^. Data for stishovite plotted on the Breit-Rabi diagram^16^,^17^ are shown in Figure 3.

Experimental results at TRIUMF are summarized in Table 1. In both powder and pellet samples of stishovite, the muon fraction was small and decreased with increasing temperature. Also, A/h's are the same for the two samples as mentioned above. On the other hand, some differences are found; the muon fraction and the relaxation rate of muonium-spin polarization were smaller in powder than in pellet. In this study, experiments were also conducted at RIKEN/RAL (UK), PSI (Switzerland), and MLF/J-PARC (Japan). Preliminary results at these facilities, such as the muon fraction and A/h in stishovite powder (roughly estimated based on decoupling measurements under longitudinal field), do not contradict those at TRIUMF described above. Experimental results for quartz are consistent with previous work^6^,^7^,^8^,^10^.

**Figure 1:**
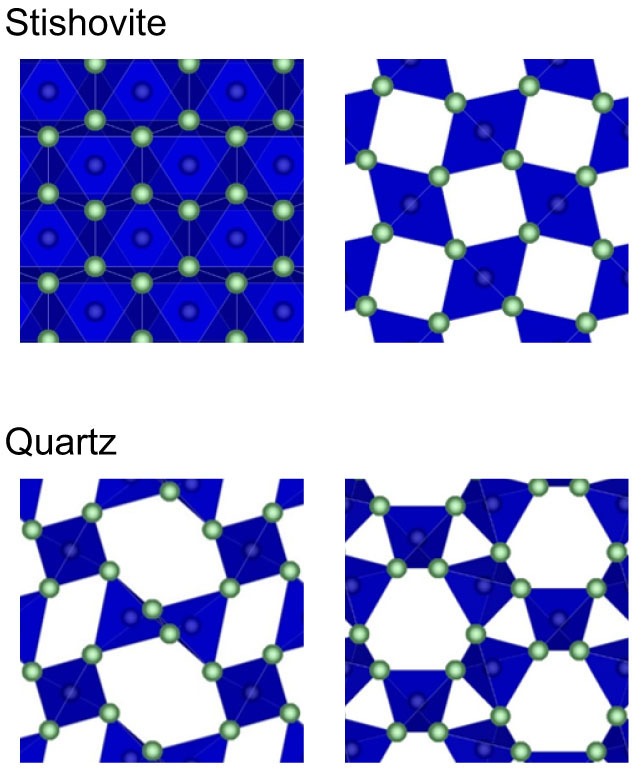
Crystal structures of stishovite and quartz. They are archetypal high-pressure six-fold coordinated and low-pressure four-fold coordinated minerals having the same chemical formula of SiO_2_. View from the direction of crystallographic a-axis (left) and c-axis (right) is shown for the both structures.

A large muonium fraction was found in stishovite as is the case in quartz. Interstitial voids of stishovite, which consists of SiO_6_ octahedra, are much smaller than those of quartz, which consists of SiO_4_ tetrahedra (Fig. 1). Therefore, the present results suggest that the formation of muonium is not controlled by the size of interstitial voids. In stishovite, not only the muonium fraction was large but also the muon fraction decreased with increasing temperature, suggesting that implanted muons become more stable by capturing an electron to form muonium than by staying in diamagnetic state. Hyperfine-coupling parameter is a measure of 1s electronic orbital size; the cube of Bohr radius is inversely proportional to this parameter^16^,^17^. Therefore, a very large hyperfine-coupling parameter of muonium in stishovite, which is even larger than that in quartz, suggests that muonium is squeezed in small interstitial voids without binding to silicon or oxygen. A very large relaxation rate of muonium-spin polarization cannot be explained by the effect of nuclear magnetic moments of silicon and oxygen because they are small. It may be due to anisotropy in hyperfine interactions. This explanation seems plausible because the relaxation rate becomes larger at low temperatures where the mobility of muoniums becomes smaller. In fact, interstitial voids in stishovite are largely anisotropic (i.e., having channels along the c-axis; Fig. 1).

The difference in muonium fraction and relaxation rate of muonium-spin polarization between powder and pellet suggests that they are affected by the grain size and/or surface condition (= defects, in a broad sense) of samples. The pellet sample is a sintered body of nanocrystals and therefore has a much smaller grain size than the powder sample. A negative correlation between grain size and muon fraction may be explained by the inhibition of delayed-muonium formation, in which positive muons capture radiolysis electrons and become muoniums after time = 0 (Ref. 10). In quartz single crystal, it has been reported that muon and muonium fractions were 20% and 80%, respectively, and about the half of muonium was formed promptly after time = 0 (as delayed muoniums)^10^. On the other hand, in quartz powder, the muon fraction was 28% (Table 1). Therefore, a negative correlation between grain size and muon fraction is also seen in quartz. The delayed formation of muonium substantially (but very shortly) after time = 0 causes the de-phasing of spin polarization. This may explain the missing fraction in powder and pellet samples; i.e., the sum of muon and muonium fractions is smaller than 100% (Table 1). At low temperatures, the muon fraction becomes larger probably because the mobility of radiolysis electrons becomes smaller and therefore the formation of delayed muonium is inhibited. In any case, muonium seems to be more easily formed at higher temperatures and/or in more perfect crystals of stishovite.

## Discussion

In this study, μSR experiments have suggested that the neutral atomic hydrogen (corresponding to muonium) can exist in stishovite without having a direct relation to lattice defects. So, the question is whether invisible hydrogen really exists in stishovite, i.e., without being detected by infrared absorption, Raman scattering, and other standard methods. Although proving the existence is difficult, a clue can be found in a report on MOS devices^19^. Infrared absorption due to Si-OH and Si-H oscillations was detected in γ-ray irradiated SiO_2_ films of MOS devices, while it was not detected in non-irradiated films. It has been interpreted as a sign that H and/or H_2_ released from an electrode of the device (hydrogen source) exist in the film and react with dangling bonds of silicon and oxygen generated by the γ-ray irradiation. Therefore, we believe that invisible hydrogen exists similarly in stishovite if hydrogen sources are available. However, even in the presence of hydrogen sources, oxidizing environment would favor the incorporation of water into stishovite by a substitution of Si^4+^ → 4H^+^ (Ref. 5). The situation may be similar in other high-pressure silicates. In previous studies of earth science, oxidizing environment has often been assumed and many experiments have been conducted in various silicate-water systems. However, the redox state of the deep mantle (from past to present) is a challenging issue^20^,^21^,^22^,^23^ and it may be much more reducing compared to the present shallow mantle^20^,^21^,^22^.

Now we assume reducing environment in the deep mantle. Then, the question is whether hydrogen sources are available in the Earth's interior. A candidate may be the core. In some models of early earth evolution, a tremendous amount of hydrogen dissolved into the core^2^,^24^. Or, hydrogen may have dissolved directly into the mantle at an early stage and stay there quietly^20^,^21^,^22^; the chemoaffinity between silicates and liquid H_2_ under high pressure has been demonstrated recently^25^. Therefore, it is possible that the neutral atomic hydrogen which is not directly related to lattice defects of silicates may exist in the mantle. To further test the hypothesis, μSR experiments should be conducted on more realistic mantle minerals and rocks. In-situ experiments under high pressure and high temperature are also indispensable. Definitely, studies from multiple viewpoints are required to know how much hydrogen can dissolve into silicates in a thermodynamically stable state. For example, accurate calculation of the formation enthalpy should be made with the help of knowledge obtained by experimental work. Neutral atomic hydrogen in the mantle will be an important research target in future earth science, because the existence form of this important element has a fundamental importance in physicochemical properties of mantle minerals, thus controls the dynamics and evolution of our planet.

## Methods

### Sample preparation

μSR spectroscopy requires a relatively large amount of samples. So, nominally anhydrous stishovite samples were synthesized under high pressure and high temperature with a belt-type large-volume press^26^ at National Institute for Materials Science (Japan) and with a Kawai-type large-volume press^27^ at Geodynamics Research Center of Ehime University (Japan), respectively. To clarify intrinsic properties of stishovite, two kinds of samples were prepared; powder sample was synthesized with a standard technique at the former institute and pellet sample (sintered nanocrystals)^28^ was synthesized with a recently developed technique at the latter institute. Stishovite powder of about 0.5 g was synthesized from noncrystalline SiO_2_ powder (Kanto Chemical Co., Inc., purity 99.9%) at 10 GPa and 1373 K with the former press. For reference in μSR experiments, quartz powder was also synthesized from the same starting material at 2 GPa and 1373 K with the same type of press. Each synthetic powder was packed in a capsule made of aluminum foil (having a thickness of 12 μm). Stishovite pellet (sintered nanocrystals) of 5.5 mm in diameter and 1.0 mm in thickness was synthesized with the latter press. More detailed information on the stishovite pellet has been given elsewhere^28^.

### Experimental procedure

μSR experiments were conducted for stishovite and quartz with surface-muon beams at RIKEN/RAL (UK), PSI (Switzerland), MLF/J-PARC (Japan), and TRIUMF (Canada). Spin-polarized positive muons were implanted into the capsulated powder or pellet and subsequent behaviors (changes in spin polarization with time) were measured under transverse magnetic field (perpendicular to the initial spin-polarization direction, up to 1000 G) or longitudinal magnetic field (parallel to the initial spin-polarization direction, up to 4000 G) at temperatures between 2.5 and 300 K (= room temperature). Stishovite powder was measured at all the four facilities and stishovite pellet was measured only at TRIUMF. Quartz powder was measured at MLF/J-PARC and TRIUMF. Based on preliminary results at RIKEN/RAL, PSI, and MLF/J-PARC, experiments with a high timing resolution, which is suitable for the observation of muonium, were conducted by using a continuous beam and a spectrometer called HiTime at TRIUMF.

### Analytical procedure

Standard procedure was followed to analyze μSR data^16^,^17^. It is briefly summarized below.

First, data obtained with HiTime under transverse magnetic field were analyzed in frequency domain. Angular frequencies of muon-spin polarization ω_μ_ and muonium-spin polarization ω_Mu1_ and ω_Mu2_ were determined by the Fourier transform. Because of the splitting of the triplet state, two frequencies appear for muonium. Thus determined ω_μ_ and gyromagnetic ratio of muon γ_μ_ were used to determine the transverse magnetic field H actually applied to the sample with the equation
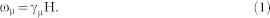
Hyperfine-coupling parameter of muonium A/h relates to ω_Mu1_ and ω_Mu2_ with the following equations;
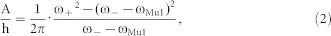

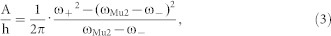

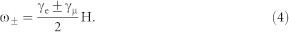
Here, h is the Plank constant and γ_e_ is the gyromagnetic ratio of electron. All the sets of (H, ω_Mu1_) and (H, ω_Mu2_) were used to determine A/h with a least-square method taking uncertainties in angular frequencies into account.

Next, data at 350 G (nominal) were analyzed in time domain with angular frequencies obtained by the Fourier transform. The equation
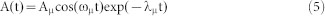
was fitted to the data at time = 0.01 ~ 2.00 μsec to determine amplitude A_μ_ and relaxation rate λ_μ_ of muon-spin polarization. Then, with fixed A_μ_ and λ_μ_, the next equation was fitted to the data at time = 0.005 ~ 0.050 μsec to determine amplitudes A_Mu1_ and A_Mu2_ and relaxation rate λ_Mu_ of muonium-spin polarization;
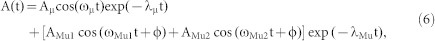
where
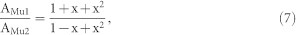

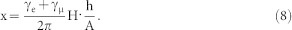
Here, phase ϕ was introduced to correct the error in time = 0. The difference in relaxation rates of two frequencies was assumed to be negligible. Then, muon fraction f_μ_ and muonium fraction f_Mu_ were determined with the equations
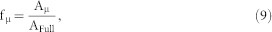

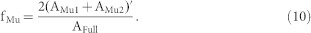
Here, A_Full_ is the amplitude for fully spin-polarized muons, which was separately calibrated with Ag as a standard. ()' denotes that quantities in the parenthesis are corrected for the error in time = 0.

**Figure 2:**
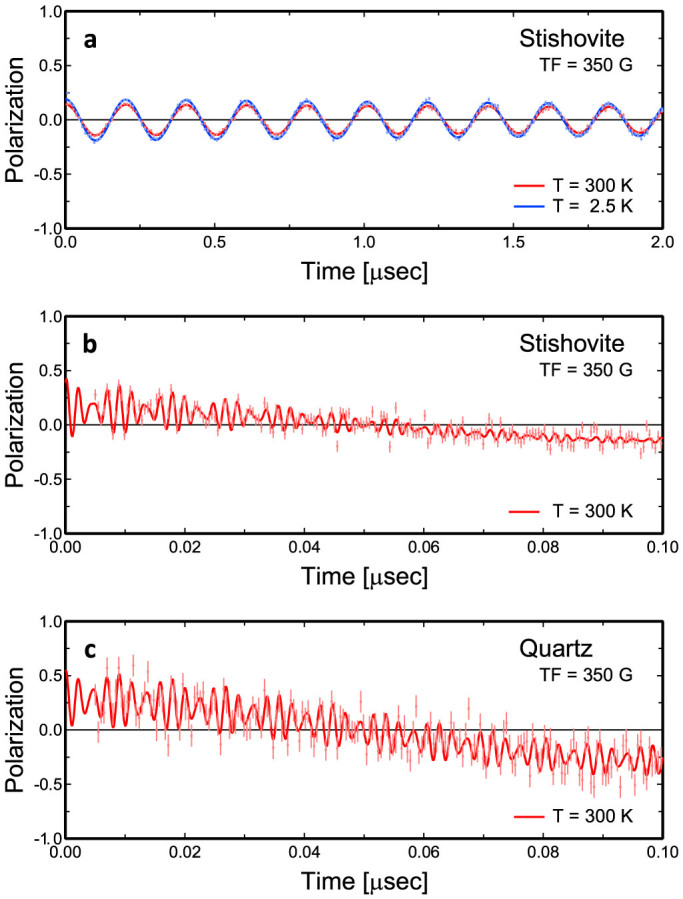
Time evolution of muon- and muonium-spin polarization in stishovite and quartz. P(t)'s are plotted (Eq. 11). (a) Muon spin in stishovite powder taken at 300 K and 2.5 K under a transverse field of 350 G (nominal). (b and c) Muonium spin in stishovite and quartz powders. The spectrum at 300 K in (a) is enlarged in terms of the time axis and is shown again in (b) for stishovite. The vertical bars represent the statistical error (standard deviation) of each point.

**Figure 3:**
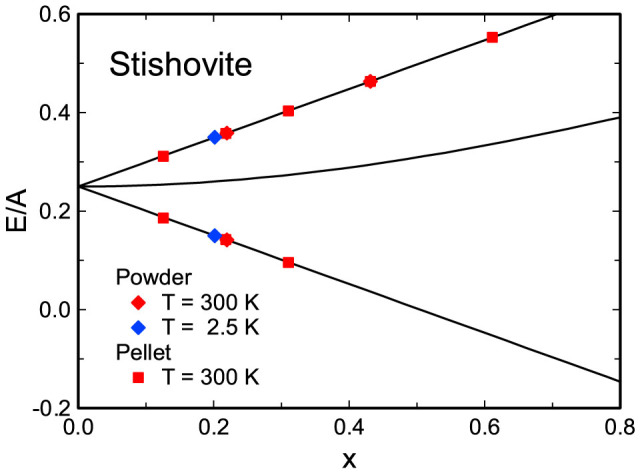
Energy diagram of muonium in stishovite as a function of applied transverse field, so called Breit-Rabi diagram^16^,^17^. x denotes the transverse field that is normalized by the field corresponding to A/h (Eq. 8). The three solid lines, in order from top to bottom, represent theoretical E_1_/A, E_2_/A, and E_3_/A (Eqs. 12–14). The data of E_1_/A and E_3_/A, from left to right, correspond to the measurements at 200, 350, 500, 700, and 1000 G (nominal). Because of a larger A/h at 2.5 K than at 300 K, the data for 2.5 K are located at the left side of corresponding data at 300 K.

In Figures 2 and 3, time evolution of spin polarization P(t) and muonium ground-state energies E_1_ and E_3_ given below are presented.
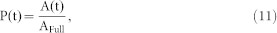
and
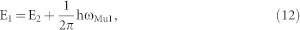

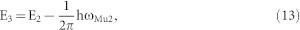
where
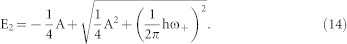


## Author Contributions

N.F., K.M.K., D.W., T.S., A.K. and R.K. designed the study. T.T., N.N. and T.I. prepared the samples. N.F., K.M.K., D.W., T.S., D.T., T.M., M.M., M.H., A.K. and R.K. conducted the μSR experiments and analyzed the data. N.F., K.M.K., D.W., T.S. and R.K. wrote the manuscript and others gave comments to improve the manuscript.

## Figures and Tables

**Table 1:** Summary of μSR measurements for stishovite^a^

Material	Form	Temperature [K]	Statistics [events]	f_μ_ [%]	λ_μ_ [μs^−1^]	f_Mu_ [%]	λ_Mu_ [μs^−1^]	A/h [GHz]
Stishovite	Powder	300	100 M	14.4(3)	0.10(1)	56(5)	24(4)	4.65(2)
		2.5	100 M	19.2(4)	0.13(1)	–b	–b	5.17(14)
	Pellet^c^	300	100 M	32.1(5)	0.05(1)	55(9)	55(9)	4.68(5)
		2.5	25 M	39.6(8)	0.06(1)	–	–	–
Quartz	Powder	300	25 M	28.0(6)	0.01(1)	56(8)	7(5)	4.49(2)

^a^f_μ_: fraction of muon, λ_μ_: relaxation rate of muon, f_Mu_: fraction of muonium, λ_Mu_: relaxation rate of muonium, A/h: hyperfine-coupling parameter of muonium, where h is the Plank constant (see Methods for more details). Measurements shown in Figure 3 were used to determine A/h for stishovite and measurements at 350 and 700 G (nominal) were used to determine A/h for quartz. Measurements at 350 G (nominal) were used to determine the other parameters.

^b^Although the simultaneous determination of f_Mu_ and λ_Mu_ was not successful due to the very fast relaxation, the relation between the two parameters can be expressed well as f_Mu_ = 0.83λ_Mu_ + 8.1.

^c^Sintered nanocrystals (Ref. 28).
